# Retraction: miR-101 targeting ZFX suppresses tumor proliferation and metastasis by regulating the MAPK/Erk and Smad pathways in gallbladder carcinoma

**DOI:** 10.18632/oncotarget.28786

**Published:** 2025-12-31

**Authors:** Run-Fa Bao, Yi-Jun Shu, Yun-Ping Hu, Xu-An Wang, Fei Zhang, Hai-Bin Liang, Yuan-Yuan Ye, Huai-Feng Li, Shan-Shan Xiang, Hao Weng, Yang Cao, Xiang-Song Wu, Mao-Lan Li, Wen-guang Wu, Yi-Jian Zhang, Lin Jiang, Qian Dong, Ying-Bin Liu

**Affiliations:** ^1^Department and laboratory of General Surgery, Xinhua Hospital, Affiliated with Shanghai Jiao Tong University, School of Medicine, No. 1665 Kongjiang Road, Shanghai 200092, China; ^2^Institute of Biliary Tract Disease, Shanghai Jiao Tong University School of Medicine, No. 1665 Kongjiang Road, Shanghai 200092, China; ^3^These authors have contributed equally to this work

**This article has been retracted:** Oncotarget has concluded its investigation of this paper. The investigation revealed multiple duplications and manipulations of images within the article. Specifically, we found the following issues:

- Figure 3A, Invasion assay: panel “NOZ: miR-NC” was duplicated in Figure 5F, panel “NOZ: miR-NC+Vector.”
- Figure 3A, Invasion assay: panel “GBC-SD: miR-NC” was duplicated in Figure 6C, panel “GBC-SD: TGF-b 48 h.”
- Figure 3A, Invasion assay: panel “GBC-SD: miR-101” was duplicated in Figure 7A panel “GBC-SD: miR-101-TGF-b.”
- Figure 5F, Migration assay: panel “NOZ: miR-NC+Vector” was duplicated in Figure 6C, panel “NOZ: TGF-b 48 h.”
- Figure 5F, Invasion assay: panel “GBC-SD: miR-NC+Vector” was duplicated in Figure 6C, panel “GBC-SD: TGF-b 48 h.”
- Figure 5G, Wound healing assay, panel “NOZ: 48 h: miR-NC+Vector” was duplicated in Figure 7B, panel “NOZ: -TGF-b-miR-101 48 h.”
- Figure 7C, Western blots: the panel labeled “B-catenin” has been horizontally reversed and used as the panel labeled “Vimentin.”

While the authors provided corrected versions of Figures 3A, 5F, 5G, 6C and 7C, the unmodified images from the original experiments were not supplied for all figures to support these corrections. Due to the substantial number of duplications in the manuscript, and the absence of original supporting data, the Editorial decision has been made to retract this paper. We attempted to contact the authors regarding this decision but received no response.

Original article: Oncotarget. 2016; 7:22339–22354. 22339-22354. https://doi.org/10.18632/oncotarget.7970

